# Transcription Factor LjWRKY50 Affects Jasmonate-Regulated Floral Bud Duration in *Lonicera japonica*

**DOI:** 10.3390/plants14152328

**Published:** 2025-07-27

**Authors:** Yanfei Li, Yutong Gan, Guihong Qi, Wenjie Xu, Tianyi Xin, Yuanhao Huang, Lianguo Fu, Lijun Hao, Qian Lou, Xiao Fu, Xiangyun Wei, Lijun Liu, Chengming Liu, Jingyuan Song

**Affiliations:** 1 State Key Laboratory of Bioactive Substance and Function of Natural Medicines, Key Lab of Chinese Medicine Resources Conservation, State Administration of Traditional Chinese Medicine of the People’s Republic of China, Engineering Research Center of Chinese Medicine Resource, Ministry of Education, Institute of Medicinal Plant Development, Chinese Academy of Medical Sciences & Peking Union Medical College, Beijing 100193, China; yfli@implad.ac.cn (Y.L.); keira1106@163.com (Y.G.); qiguihong12@163.com (G.Q.); wjxu@implad.ac.cn (W.X.); xintianyi87@sina.com (T.X.); huangyuanhao@implad.ac.cn (Y.H.); fulianguo99@foxmail.com (L.F.); haolj_cpu@foxmail.com (L.H.); louq97@126.com (Q.L.); 2 Shandong Honeysuckle Association, Linyi 273300, China; sdjyhxh@126.com (X.F.); 13053932155@163.com (X.W.); 3 Pingyi County Longdingshan Farm Co., Ltd., Linyi 273300, China; cnjiujianpeng@163.com (L.L.); 13953909766@139.com (C.L.)

**Keywords:** *Lonicera japonica*, floral bud duration, jasmonate, resequencing

## Abstract

*Lonicera japonica* Thunb. is a traditional Chinese medicinal herb whose floral buds are the primary source of pharmacological compounds that require manual harvesting. As a result, its floral bud duration, determined by the opening time, is a key determinant of both quality and economic value. However, the genetic mechanisms controlling floral bud duration remain poorly understood. In this study, we employed population structure analysis and molecular experiments to identify candidate genes associated with this trait. The improved cultivar Beihua No. 1 (BH1) opens its floral buds significantly later than the landrace Damaohua (DMH). Exogenous application of methyl jasmonate (MeJA) to BH1 indicated that jasmonate acts as a negative regulator of floral bud duration by accelerating floral bud opening. A genome-wide selection scan across 35 germplasms with varying floral bud durations identified the transcription factor *LjWRKY50* as the causative gene influencing this trait. The dual-luciferase reporter assay and qRT-PCR experiments showed that LjWRKY50 activates the expression of the jasmonate biosynthesis gene, *LjAOS*. A functional variant within *LjWRKY50* (Chr7:24636061) was further developed into a derived cleaved amplified polymorphic sequence (dCAPS) marker. These findings provide valuable insights into the jasmonate-mediated regulation of floral bud duration, offering genetic and marker resources for molecular breeding in *L. japonica*.

## 1. Introduction

*Lonicera japonica* Thunb. (*L. japonica*), commonly referred to as Jinyinhua, is valued both for its ornamental appeal due to its fragrant, showy flowers, and also for its ecological role in soil and water conservation [1,2]. In addition to these uses, *L. japonica* is a widely consumed medicinal and edible plant. Its flowers are frequently used in traditional Chinese medicine (TCM) and culinary and health-related products [3,4]. Pharmacological research has shown that *L. japonica* contains a variety of bioactive compounds, including organic acids, volatile oils, and flavonoids, with demonstrated antiviral, antioxidant, antibacterial, and immunomodulatory effects [3,5,6]. According to the Chinese Pharmacopoeia (2020 Edition), only unopened or newly opened floral buds are suitable for medicinal use. Thus, the floral bud’s duration, particularly the period it remains unopened, is vital for determining its medicinal quality. Investigating the factors that influence flower opening in *L. japonica* is therefore of both practical and scientific importance.

Flower development in *L. japonica* progresses through seven stages, going from young bud to senescence: juvenile floral bud (S1), three-green floral bud (S2), two-white floral bud (S3), complete white floral bud (S4), silver flower (S5), gold flower (S6), and tawny withering flower (S7) [2,7]. The S3 and S4 stages are considered optimal for harvest, both visually and chemically, but this window typically lasts only two days [8,9]. The brief duration limits harvest efficiency, which in turn affects the quality and consistency of the final product. To address this, long-duration floral bud cultivars have been developed to enable more centralized harvesting, reduce labor costs, and enhance the grade of medicinal materials. One such cultivar, Beihua No. 1 (BH1), remains in the S4 stage for over one week and accumulates high concentrations of active compounds [10,11]. Yet, the genetic mechanisms underlying the prolonged bud duration in BH1 remain unclear.

Bud duration is controlled by the timing of floral opening, a developmental milestone influenced by internal signals and environmental cues [12]. Jasmonate, a plant hormone, has been shown to play a regulatory role in the opening of flowers. For instance, in *Eustoma grandiflorum* (Raf.) Shinn., treatment with 100 µM methyl jasmonate (MeJA) accelerates flower opening by loosening petal cell walls via upregulation of expansin and xyloglucan endotransglycosylase/hydrolase (XTH) genes [13]. In tomato, jasmonic acid promotes flower opening through the upregulation of the transcription factor SlMYB21 [14]. In rice, jasmonate alters the timing of floret opening by promoting cell expansion in the lodicules, a process mediated through the transcription factor OsMYC2, which activates *allene oxide synthase* (*OsAOS1*) and *OsXTH9* [15,16]. In *Cucurbita pepo*, the regulation of flower opening appears to involve crosstalk between jasmonate and ethylene, although the exact mechanisms remain unknown [17]. However, the role of jasmonate in controlling flower opening in *L. japonica* remains unclear.

In this study, we examined 35 *L. japonica* germplasms to investigate the genetic basis of floral bud duration and its relationship to jasmonate signaling. Application of exogenous MeJA confirmed that jasmonate negatively regulates floral bud duration by accelerating the opening process. Population structure analysis revealed that floral bud duration was a key differentiation among subpopulations. Through whole-genome resequencing, we identified the WRKY transcription factor LjWRKY50 as a regulator of jasmonate biosynthesis, playing a crucial role in controlling floral bud duration. Additionally, we developed a derived cleaved amplified polymorphic sequence (dCAPS) marker (Chr7:24636061) within *LjWRKY50* for marker-assisted selection (MAS). These results provide both mechanistic insight into floral bud duration regulation and practical tools for breeding *L. japonica* varieties with prolonged floral bud durations.

## 2. Results

### 2.1. Exogenous MeJA Accelerates Floral Bud Opening in L. japonica

**Figure 1:**
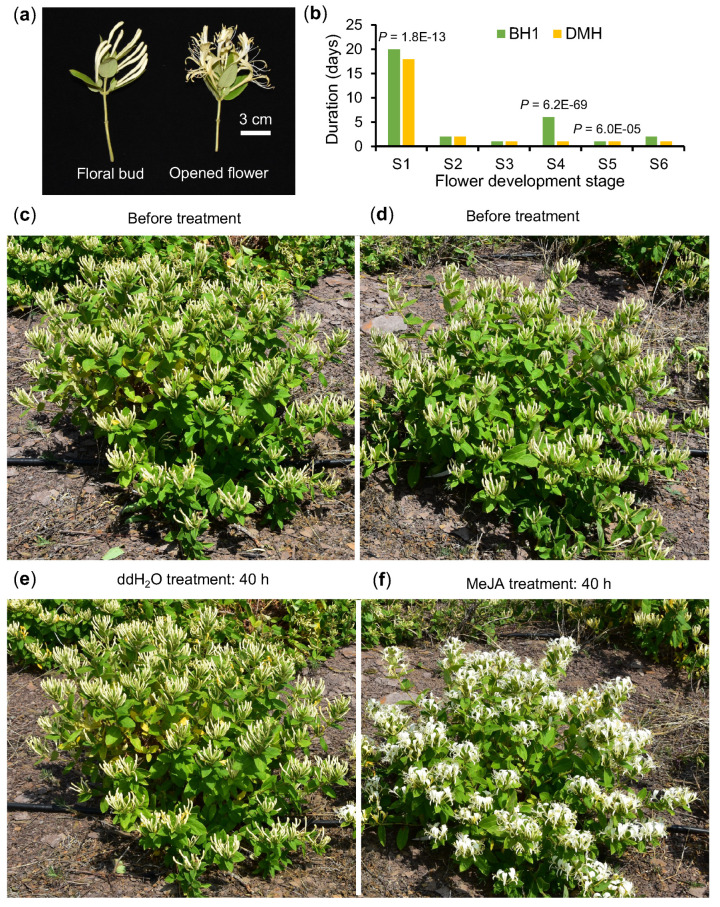
Exogenous MeJA accelerates floral bud opening in BH1. (**a**) The morphological characterization of the floral bud and the opened flower. (**b**) Statistics of flower development stages in ddH_2_O and MeJA treatments. Significant differences are shown by *p* values, using *t*-tests. (**c**,**d**) BH1 plants before treatment. (**e**) floral buds opened after ddH_2_O treatment. (**f**) floral buds opened after MeJA application (8 mmol/L).

Previous studies in rice and tomato have shown that jasmonate positively regulates flower opening [14,15]. To study whether this is also the case in *L. japonica*, we performed exogenous phytohormone experiments. The improved *L. japonica* cultivar, BH1, exhibits a prolonged complete white floral bud (S4) stage, lasting over one week. This duration is much longer than that of traditional varieties like the landrace Damaohua (DMH), which stays at S4 for only about one day (Figure 1b).

To test the effect of jasmonate, we conducted an experiment in the field by applying exogenous methyl jasmonate (MeJA). BH1 plants were sprayed with either MeJA or ddH_2_O between 5:00 and 7:00 p.m., with the concentration of MeJA set at 8 mmol/L, as referenced in experiments on rice [16]. As shown in Figure 1, floral buds at the S4 stage treated with MeJA began to open within 1.5 days (Figure 1d,f), which was significantly faster than the water-treated control, which opened within 5.5 days (Figure 1c,e). The findings indicate that MeJA accelerates floral bud opening in *L. japonica*, thereby shortening the duration of the unopened bud stage.

### 2.2. Prolonged Floral Bud Duration Trait Is Preferred in Artificial Breeding

To explore the genetic basis of floral bud duration, we conducted whole-genome resequencing on 35 *L. japonica* germplasms and three additional *Lonicera* species (Table S1). Each germplasm generated approximately 38.25 GB of raw data. The sequencing quality was consistently high, with an average Q20 score of 98.03%. Mapping rates and genome coverage averaged 99.31% and 88.60%, respectively, with all samples achieving > 30× depth (Table S1). Minor differences in mapping rates were probably caused by genetic background divergence among these germplasms.

**Figure 2:**
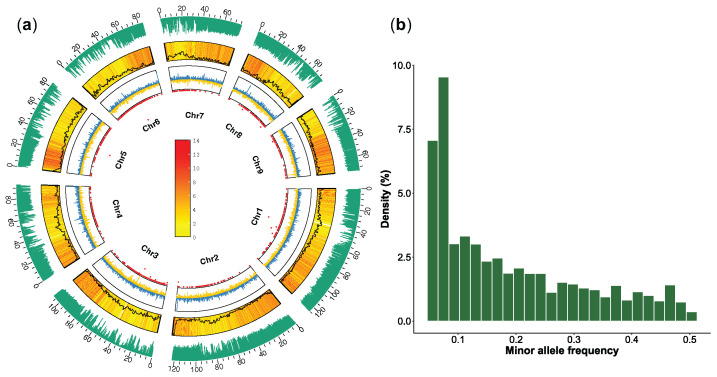
High-depth whole-genome resequencing of 35 *L. japonica* germplasms and three other *Lonicera* species. (**a**) Circos plot summarizing resequencing data: N-ratio distribution (point plot), GC skew (line plot), gene density and GC variation (heatmap and line plot), and SNP variant density (bar plot). (**b**) Minor allele frequency spectrum across the genome.

After variant calling, we identified approximately 66 million SNPs across the 38 samples. Following quality filtering (missing rate ≤ 10%, MAF ≥ 0.05), a total of 18,048,197 high-quality SNPs were retained for population genetic analysis (Figure 2a). The site frequency spectrum (SFS) displays an almost even distribution of those SNPs, ensuring sufficient coverage and high resolution for genetic population analyses (Figure 2b). This analysis represents the largest SNP dataset to date for *L. japonica*, which is publicly accessible at https://ngdc.cncb.ac.cn under PRJCA034608, providing a valuable resource for future breeding and association studies.

**Figure 3:**
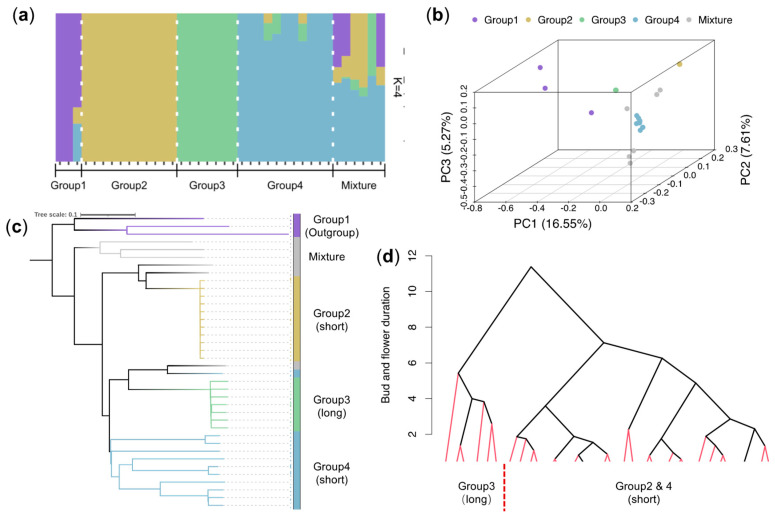
Population structure and trait distribution of 35 *L. japonica* germplasms and three other *Lonicera* species. (**a**) ADMIXTURE analysis at K = 4. (**b**) Principal component analysis (PCA). (**c**) Neighbor-joining tree based on SNP data. (**d**) Dendrogram showing floral bud and flower duration across *L. japonica* germplasm.

To investigate genetic relationships and structure, we applied ADMIXTURE [18], principal component analysis (PCA) [19], and rooted neighbor-joining tree construction [20]. A total of 35 *L. japonica* germplasms and three other *Lonicera* species that were set as outgroups (*L. tellmanniana* Spaeth, *L. macranthoides* Hand.-Mazz., and *L. hypoglauca* Miq.) were included. The 35 *L. japonica* germplasms were collected from Shandong (116.75 ~ 118.40° E, 35.08 ~ 36.55° N), Henan (114.42° E, 35.04° N), and Hebei (115.04 ~ 116.87° E, 37.22 ~ 38.31° N), China. A set of 18,048,197 SNPs obtained from resequencing and filtering across the whole genome of 38 *Lonicera* samples was utilized for population structure analyses. ADMIXTURE assigned the 38 samples into four genetic groups at K = 4, with a threshold of 80% ancestry for group assignment (Figure 3a and Figure S1). Group 1 included the three non-japonica species (*L. tellmanniana* Spaeth, *L. macranthoides* Hand.-Mazz., and *L. hypoglauca* Miq.), serving as the outgroup. Among the *L. japonica* samples, Group 2 contained 11 samples, Group 3 contained 7, and Group 4 included 11. Six samples exhibited mixed ancestry, indicating possible gene flow resulting from hybridization, introgression, or natural mutation (Table S2). PCA results showed that the samples within each group were clustered together (Figure 3b). The phylogenetic tree indicated that the same group was located on the same branch (Figure 3c). These results support the interpretation of convergence across the three methods, suggesting the presence of a structured population.

As a typical clonally propagated plant, the overall nucleotide diversity (π) across *L. japonica* germplasms was 5.51 × 10^−3^, which was higher than that observed in other clonally propagated plants such as cassava (π = 1.8 × 10^−3^), indicating substantial genetic diversity in this species (Figure S2). Notably, Group 4 exhibited the highest dispersion in PCA and genetic distance in the phylogenetic tree, suggesting greater internal variation (Figure S2). We further calculated π values for each subpopulation: Group 4 had the highest diversity (π = 5.22 × 10^−3^), nearly twice that of Group 2 (π = 2.85 × 10^−3^) and Group 3 (π = 2.18 × 10^−3^), implying that Group 2 and Group 3 may have experienced stronger artificial selection during evolution.

### 2.3. LjWRKY50 Is Associated with the Prolonged Duration of Floral Buds

Germplasm resources with favorable phenotypic traits that undergo positive selection during domestication or breeding often show reduced nucleotide diversity (π). This pattern has been used to identify candidate genes under selection in species such as soybean [21,22] and maize [23]. Long-duration floral bud germplasms have been developed through artificial breeding in recent years. To explore whether floral bud duration in *L. japonica* has been shaped by artificial selection, we compared polymorphism levels between long- and short-duration floral bud groups using our SNP dataset.

Population structure analysis divided the 35 *L. japonica* samples into three groups, with Group 3 clearly separated from Groups 2 and 4 due to its prolonged floral bud duration (Figure 3a–c). Hierarchical clustering based on floral bud duration phenotype further supported this, dividing the germplasms into two main branches representing long and short durations (Figure 3d). These results indicate that prolonged floral bud duration was probably a major trait selected during artificial breeding.

**Figure 4:**
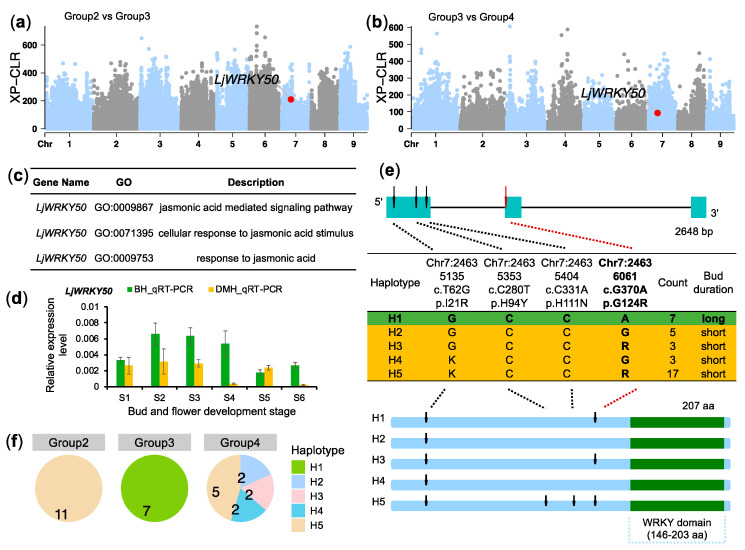
LjWRKY50 is associated with floral bud duration. (**a**,**b**) XP-CLR scans showing selective sweeps between Group 2 vs. Group 3 (**a**) and Group 3 vs. Group 4 (**b**). (**c**) GO terms associated with *LjWRKY50*. (**d**) The relative expression profiles of *LjWRKY50* in BH1 and DMH, with the *LjActin* as the reference gene. (**e**) LjWRKY50 gene structure (**top**), protein-altering variants and their locations (**middle**), and protein domain layout (**bottom**) across seven haplotypes. (**f**) Distribution of LjWRKY50 haplotypes across Groups 2, 3, and 4.

We applied the cross-population composite likelihood ratio (XP-CLR) method to identify selective sweeps caused by empirical selection. XP-CLR values were calculated for comparisons between Group 3 and Group 2, as well as between Group 3 and Group 4, using 100 kb slicing windows with 10 kb steps (Figure 4a,b). The top 5% of XP-CLR scores were used as a threshold to define selective sweeps. This approach identified 103 candidate regions spanning 9.02 Mb, which contain 213 annotated genes (Table S3). These genes are potential targets for artificial selection due to the prolonged duration of the floral bud.

Given the role of jasmonate in floral bud opening, we screened the 213 candidate genes for those linked to jasmonate signaling or metabolism. Three genes were identified. Of these, *Lj5A256T13* encodes the flavin-binding kelch repeat F-box protein 1 (FKF1), and responds to jasmonic acid (GO:0009753) according to Gene Ontology Analysis; *Lj7C246T2* encodes the WRKY transcription factor 50 (WRKY50), involved in jasmonic acid mediated signaling pathway (GO:0009867), cellular response to jasmonic acid stimulus (GO:0071395), and response to jasmonic acid (GO:0009753) (Figure 4c); and *Lj8A490G27*, which is a homolog of methyl jasmonate esterase 1 (MJE1) that is involved in the jasmonic acid metabolic process (GO:0009694).

Regarding our unpublished data, the concentrations of MeJA increased at the S5 and S6 stages in BH1, indicating that the jasmonate biosynthesis pathway should be complete. Thus, the transcription factor LjWRKY50, whose homolog activates the jasmonate acid biosynthetic gene *AOS* in tomato [24], was identified as the candidate gene responsible for regulating floral bud duration by modulating jasmonate biosynthesis.

### 2.4. LjWRKY50 Influences Jasmonate Biosynthesis in L. japonica

To confirm the role of *LjWRKY50* in regulating floral bud duration, we first performed homologous protein sequence comparison analysis and qRT-PCR experiments. A comparison of putative protein sequences in the NCBI database identified a conserved WRKY domain spanning amino acids 146 to 203 in LjWRKY50 (Figure S3). The gene expression level of *LjWRKY50* in BH1 was similar to that in DMH, suggesting that the functional variants were not situated in its promoter region (Figure 4d). Given that LjWRKY50 encodes a transcription factor (TF), we speculated that variations in the coding region may affect its TF-DNA binding ability, which in turn shapes the expression of the downstream gene [25,26,27].

We examined sequence data from the 35 resequenced germplasms and identified four nonsynonymous variants in *LjWRKY50*, forming five distinct haplotypes (Table S4). Haplotype H1 (*LjWRKY50^H1^*) was found exclusively in germplasms with long floral bud durations, while haplotypes H2–H5 were associated with short-duration types (Figure 4e). The nonsynonymous variant Chr07:24636061, which causes an amino acid change from G to R, was closely linked to floral bud duration. Alleles Chr07:24636061-A and Chr07:24636061-G represent long- and short-duration floral buds, respectively. Therefore, Chr07:24636061 is predicted to influence protein function, even though it is located outside the conserved WRKY domain (Figure 4e). This haplotype *LjWRKY50^H^^1^* was fixed in Group 3 (long-duration group), while Group 2 carried only *LjWRKY50^H5^*, indicating low diversity in both groups (Figure 4f).

**Figure 5:**
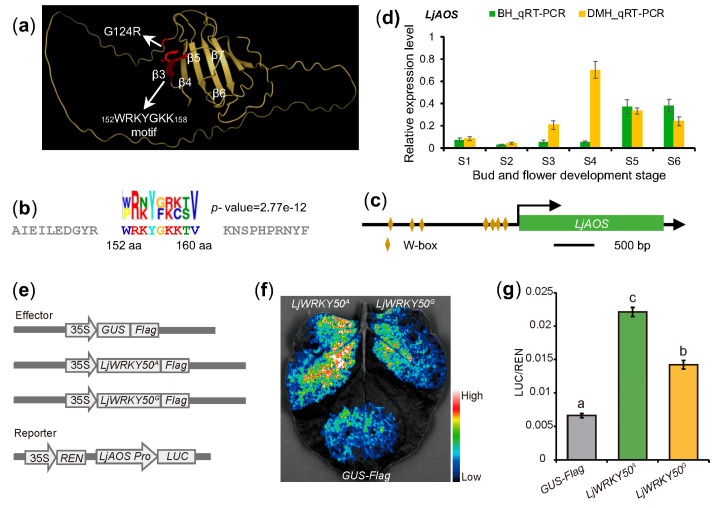
LjWRKY50 regulates jasmonate biosynthesis by binding the *LjAOS* promoter. (**a**) Predicted 3D structure of LjWRKY50 and its interaction with a W-box element. (**b**) The WRKY domain, including the conserved motif WRKYGKK. (**c**) Location of W-box cis-elements within the *LjAOS* promoter. (**d**) The relative expression profiles of *LjAOS* in BH1 and DMH (S1-S6), with the *LjActin* as the reference gene. (**e**) Schematic of the dual-luciferase reporter assay vectors in *Nicotiana benthamiana* leaves: LUC (firefly luciferase) driven by the LjAOS promoter, and REN (Renilla luciferase) under the 35S promoter. (**f**) Fluorescence images of *N. benthamiana* leaves three days after infiltration. (**g**) Quantification of relative LUC activity in samples expressing LjWRKY50^A^ or LjWRKY50^G^. Values are means ± SD from three biological replicates. Significant differences are indicated by lowercase letters (*p* < 0.01, ANOVA with Tukey’s post hoc test).

To better understand how the G124R variant might influence LjWRKY50 function, we predicted its 3D protein structure. The DNA-binding domain (DBD) of LjWRKY50 adopted a five-stranded antiparallel β-sheet conformation (β3–β7). The conserved WRKY motif (152WRKYGKK158) was located within β3 (Trp152-Lys158), which interacted with W-box motifs (cis-elements with the sequence (C/T)TGAC(C/T)) in the promoters of target genes, *LjAOS* (Figure 5a). The variant G124R was positioned near this DNA-binding motif, suggesting it may affect DNA affinity (Figure 5b). We then examined seven WRKY-binding W-box elements within the -2000 bp promoter region of *LjAOS* (Figure 5c). These clustered motifs likely facilitate the binding of LjWRKY50. Consistent with the phenomenon of jasmonate inhibiting flower opening, the expression of the jasmonate biosynthesis gene *LjAOS* was lower in BH1 (long-duration) than in DMH (long-duration) at floral development stages S3 and S4 (Figure 5d). Together, these findings support LjWRKY50’s role in regulating *LjAOS* expression, which in turn influences jasmonate levels and floral bud opening.

To confirm that LjWRKY50 regulates *LjAOS* expression in vivo, we conducted a dual-luciferase assay. Using cDNA templates from BH1 and DMH, we cloned the *LjWRKY50* and confirmed the presence of the potential functional variant Chr07:24636061, which carries either the A or G allele (Figure S4). We then assessed the transcriptional activity of LjWRKY50^A^ and LjWRKY50^G^ by co-expressing them with a firefly luciferase (LUC) reporter driven by the *LjAOS* promoter. A Renila reniformis luciferase (REN) reporter gene, under the control of the 35S promoter, served as the internal control (Figure 5e).

Both LjWRKY50^A^-Flag and LjWRKY50^G^-Flag significantly increased LUC reporter expression compared to the control Flag protein (Figure 5f,g). However, the two alleles exhibited different levels of activation, indicating that LjWRKY50 is a transcriptional enhancer of *LjAOS* and that functional differences between the A and G alleles at Chr7:24636061 influence this regulatory effect.

### 2.5. A dCAPS Marker Is Developed to Identify Germplasms with Prolonged Floral Bud Duration

**Figure 6:**
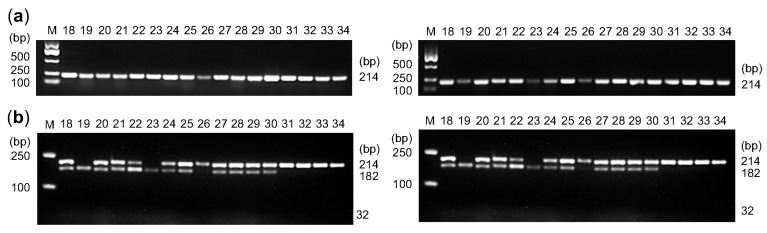
Functional analysis of LjWRKY50 alleles and development of a dCAPS marker. (**a**) Amplification products for 34 *L. japonica* germplasms, which showed as the 214 bp bands. (**b**) dCAPS genotyping results for 34 *L. japonica* germplasms. A 214 bp band indicates the Chr7:24636061-AA genotype; digestion into 184 bp and 32 bp fragments indicates the Chr7:24636061-GG genotype. All three bands indicate the Chr7:24636061-AG genotype.

We developed a derived cleaved amplified polymorphic sequence (dCAPS) marker targeting the Chr7:24636061 site and used it to genotype 34 germplasms. The amplification product was a 214 bp fragment (Figure 6a and Table S5). Samples with the Chr7:24636061-AA genotype were not cleaved by the NlaIII restriction enzyme, resulting in a single 214 bp fragment. Those with the Chr7:24636061-GG genotype were cleaved by the restriction enzyme NlaIII into two fragments of 184 bp and 32 bp. Heterozygous Chr7:24636061-AG genotypes produced all three fragments: 214, 184, and 32 bp (Figure 6b). The dCAPS marker enabled efficient identification of germplasms carrying genotypes associated with prolonged floral bud duration, offering a useful tool for marker-assisted selection in *L. japonica* breeding.

## 3. Discussion

The prolonged duration of floral buds in *L. japonica*, caused by delayed bud opening, is an artificially selected trait and seems to be unique to *L. japonica*, fulfilling a practical need. Cultivars with prolonged floral bud stages have been selectively bred due to the superior quality of unopened buds and the reduced cost associated with centralized harvesting. Despite its importance, the genetic mechanisms underlying floral bud duration have remained largely unknown. In this study, we demonstrate that jasmonate acts as a negative regulator of floral bud duration by promoting bud opening. We also suggest that the LjWRKY50-LjAOS regulatory module controls this process by affecting jasmonate biosynthesis, providing key targets for the genetic improvement of *L. japonica* cultivars.

Our findings show that jasmonate accelerates the opening of floral buds in *L. japonica*, which is consistent with previous studies in *Cucurbita pepo* and rice [16,17]. As shown in Figure 1, application of exogenous MeJA to BH1, a cultivar with characteristically late-opening flowers, triggered rapid bud opening. This result confirms that jasmonate plays a regulatory role in floral opening in *L. japonica*, highlighting its potential as a useful phytohormone for manipulating flower opening. In rice, the lodicules control floret opening through turgor changes caused by cell swelling [15]. *japonica*. Future studies examining the cellular dynamics and structural features of floral buds in *L. japonica* will be essential for uncovering the regulation mechanism of bud duration.

We identified a transcription factor, LjWRKY50, as a key regulator of *LjAOS* expression, which is involved in jasmonate biosynthesis and is thus directly linked to the timing of floral bud opening. In *A. thaliana*, the WRKY50 and WRKY51 proteins regulate low oleic acid-dependent repression of JA signaling [28]. In tomato, SlWRKY50 enhances cold tolerance by regulating JA biosynthesis, and its expression is in turn activated by SlMYC2, part of the JA signaling feedback loop [24]. These studies suggest that the WRKY family is involved in the JA pathway. Collectively, our findings suggest that *LjWRKY50* and *LjAOS* form a functional module that modulates floral bud opening in *L. japonica* via jasmonate accumulation.

Marker-assisted selection (MAS) is an efficient method for precision plant breeding [29,30,31]. Although prior studies have identified candidate genes and SSR markers in *L. japonica* [32,33], functional genes or markers directly associated with economically valuable traits, such as prolonged floral bud duration, have remained elusive. In other plants, dCAPS markers have been developed for traits such as low seed alkaloid content in narrow-leafed lupin (*Lupinus angustifolius* L.) and fruit color in pepper via the CaAPRR2-like gene [34,35]. The above reports indicate that the dCAPS marker is an effective tool for selecting favorable traits. In this study, we identified a nonsynonymous SNP at Chr7:24636061 within LjWRKY50, with A or G alleles corresponding to long or short floral bud durations, respectively. Therefore, we develop a dCAPS marker targeting this SNP to support MAS for long-duration floral buds in *L. japonica*.

Our phylogenetic tree of 35 *L. japonica* shows that individuals within Group 2 and Group 3 had identical backgrounds (Figure 3c). The same phenomenon was reported in orange [36], which is also propagated asexually through grafting. In orange, somatic variations are a major source of genetic diversity. Here, we hypothesize that somatic mutants may contribute to the closer genetic relationship within Group 2 and Group 3 in our study. Because somatic variations are a major source of phenotype diversity in asexual plants [37], they will be utilized more precisely and efficiently in future molecular breeding to develop individuals with favorable traits in *L. japonica*.

Based on these findings, we propose a mechanistic model for floral bud opening in *L. japonica*. In germplasms carrying LjWRKY50^G^, the transcription factor enhances *LjAOS* expression, leading to increased jasmonate biosynthesis and earlier floral bud opening. In contrast, the LjWRKY50^A^ variant fails to activate *LjAOS*, possibly due to interference by another protein [38], resulting in reduced jasmonate levels and delayed floral bud opening. This prolongation extends the floral bud stages, enabling centralized harvesting, reducing labor input, and enhancing economic efficiency.

## 4. Materials and Methods

### 4.1. Phenotype Investigation and Analyses

This study utilized 35 *L. japonica* germplasms, comprising 19 landraces and 16 cultivars, collected from Shandong (31), Henan (1), and Hebei (3) for selective sweep detection and haplotype analysis. Additionally, three other *Lonicera* species, *L. tellmanniana* Spaeth, *L. macranthoides* Hand.-Mazz., and *L. hypoglauca* Miq., were used as outgroups in population structure analyses. All plant materials were identified by Yulin Lin (Institute of Medicinal Plant Development, Chinese Academy of Medical Sciences & Peking Union Medical College) and Fengqin Zhou (College of Pharmacy, Shandong University of Traditional Chinese Medicine). These plants were cultivated at Longdingshan Farm Co., Ltd. in Pingyi County, China (121.50° E, 31.41° N), a region known as the Hometown of Honeysuckle.

Flower development included seven stages: juvenile floral bud (S1), three-green floral bud (S2), two-white floral bud (S3), complete white floral bud (S4), silver flower (S5), gold flower (S6), and tawny withering flower (S7) [2,7]. The stages of 35 *L. japonica* germplasms were recorded daily from the appearance of flower buds, and the duration of each stage was calculated: Stage duration (days) = Day_next stage_ (the day the next stage appears)—Day_last stage_ (the day the last stage appears). A distance matrix was generated from the duration dataset using the Euclidean method. Hierarchical clustering was performed using the ward. D2 method, and dendrograms were constructed using R packages.

### 4.2. Exogenous MeJA Treatment

For MeJA treatment, BH1 plants bearing floral buds of similar developmental stages were labeled. Based on previous studies, MeJA was applied at a concentration of 8 mmol/L [16]. Spraying was conducted daily from 5:00 to 7:00 p.m. because the floral opening time for *L. japonica* was always in the evening. using either 8 mmol/L MeJA solution (Coolaber, Beijing, China, CJ6691) or ddH_2_O as a control. The plants were sprayed until saturated. Each treatment group consisted of three plants. Bud opening was recorded daily at 5:00 p.m. following the initial spray application.

### 4.3. Whole Genome Resequencing, Read Alignment, and Variant Calling

Genomic DNA was extracted from 35 *L. japonica* germplasms and three other *Lonicera* species, using one individual per germplasm. DNA samples were quantified (>3 μg; >30 ng/μL; OD_260_/OD_280_ = 1.80–2.00) and fragmented (~350 bp) to construct sequencing libraries. Sequencing was performed on the DNBSEQ-T7 platform (MGI Tech, Shenzhen, China). Low-quality reads were filtered out if they met any of the following criteria: (1) presence of adapter sequences; (2) > 1% ambiguous bases (N); (3) quality score ≤ 10. Clean reads were mapped to the *L. japonica* reference genome (GWHAAZE00000000, Genome Warehouse—National Genomics Data Center) [2,39] using the Burrows-Wheeler Aligner (BWA) (v.0.7.15) [40]. Variants (SNP and indels) were called using GATK (v.3.4.46) [41] and filtered with the following criteria: QD < 2.0, FS > 60.0, MQ < 40.0, MQRankSum ≤ −12.5, ReadPosRankSum < −8.0, and non-biallelic SNPs. A total of 66,276,671 high-quality SNPs were retained for further analyses.

### 4.4. Population Structure Analysis

Variants with missing rates > 10% and minor allele frequency (MAF) < 5% were excluded, leaving 18,048,197 SNPs for population structure analysis. Three approaches were used. First, ADMIXTURE (v.1.3.0) [18] was used for model-based clustering, testing K = 1–10. Second, a neighbor-joining (NJ) tree was constructed from a nucleotide *p*-distance matrix using TreeBeST (v.1.9.2) with 100 bootstrap replicates [20]. Third, principal component analysis (PCA) was performed in PLINK (v.1.07) using a standardized relationship matrix [19]. The results were visualized with the scatterplot3d R package (v.0.3-44). The first three PCs were compared with each other across all 38 *L. japonica* germplasms.

### 4.5. Nucleotide Diversity and Selective Sweep Detection

Nucleotide diversity (π) was calculated using VCFtools (v.4.1) with the following command: “vcftools --keep --window-pi 100,000 --window-pi-step 10,000” [42]. Selective sweeps were identified using XP-CLR (v.1.1.2) with the parameters: “--size 100,000 -step 10,000” [43]. The top 5% of genomic regions were defined as selective sweeps, as described previously [44].

### 4.6. Quantitative Real-Time PCR

Floral buds or flowers from BH1 and DMH were collected at six developmental stages (S1–S6), with three biological replicates per stage. Total RNA was extracted using TRIzol reagent (Invitrogen), and 5 µg of RNA was reverse transcribed into cDNA using the TRAN kit. Real-time PCR (RT-PCR) was conducted using a two-step method, and gene expression was quantified using the 2^−△Ct^ method: △Ct = CT(*gene*) — CT(*LjActin*) [45]. Primer sequences are listed in Table S5. Three biological repetitions and three technical repetitions were used in the qRT-PCR.

### 4.7. Candidate Gene Analyses

The protein structure of LjWRKY50 was predicted using AlphaFold2 [46] and visualized with PyMOL v.3.05 [47]. Conserved motifs were identified with MEME (v.5.5.7) using the following command: “meme LjWRKY50 -protein -oc. -nostatus -time 14,400 -mod anr -nmotifs 1 -minw 6 -maxw 50 -objfun classic -markov_order 0” [48]. Cis-regulatory elements in the 2 kb promoter region were identified using PlantPAN 4.0.

### 4.8. Dual-Luciferase Reporter Assay

To assess the function of LjWRKY50 on *LjAOS*, a 2 kb promoter fragment of *LjAOS* was cloned into the pGreenII 0800-LUC vector to construct the reporter. The CDS of *LjWRKY50^A^* and *LjWRKY50^G^* were cloned into the JRH0641-Flag vector using XhoI sites with the In-fusion system. The JRH0641-Flag, *LjWRKY50^A^*-Flag, and *LjWRKY50^G^*-Flag plasmids were co-transformed with the *LjAOS Pro*-LUC plasmid into Nicotiana benthamiana leaves.

Luciferase activities (firefly LUC and Renilla REN) were measured using the Dual-Luciferase^®^ Reporter Assay System (Promega, Madison, WI, USA). Relative promoter activity was calculated as the LUC/REN ratio. Additionally, *N. benthamiana* leaves were infiltrated with 1 mM D-luciferin sodium salt substrate and photographed using a low-light, cooled charge-coupled device camera (Tanon 5200, Beijing, China).

## Data Availability

The whole genome resequencing data are available from the National Genomics Data Center, China National Center for Bioinformation/Beijing Institute of Genomics, Chinese Academy of Sciences, and are publicly accessible at https://ngdc.cncb.ac.cn under PRJCA034608.

## Supplementary Materials

The following supporting information can be downloaded at: https://www.mdpi.com/article/10.3390/plants14152328/s1, Figure S1: Population structure analysis of 38 *Lonicera* samples using ADMIXTURE software. (a) The cross-validation error from ADMIXTURE software. (b) Population structure from clusters one to ten. Each color and bar represents an individual population and accession, respectively. The segments of different colors represent the proportions of ancestral populations. A dashed white line divides the germplasms into five distinct clusters. Figure S2: The nucleotide diversity (π) analyses using 35 resequenced *L. japonica* germplasms. (a–c) The Manhattan plots display the nucleotide diversity (π) of Group 4, Group 3, and Group 2 across the entire genome, employing a 100 kb window and a 10 kb step, respectively. (d) The average nucleotide diversity (π) within the group. Figure S3: The alignment results of the protein sequence for WRKY50 in *L. japonica* and the other ten species. Figure S4: The alignment of CDS sequences for the *LjWRKY50* gene, which was cloned from the long and short floral bud duration varieties BH1 and DMH, involved two biological replications. Table S1: Summary information on the resequencing data for 35 *L. japonica* germplasms and three other Lonicera species. Table S2: Results of population structure analysis for 35 *L. japonica* germplasms and three other *Lonicera* species using ADMIXTRUE software. Table S3: The 213 selected genes for long floral bud duration were identified using the XP-CLR approach. Table S4: Information on haplotypes for 35 *L. japonica* germplasms and three other *Lonicera* species. Table S5: Primers’ information used in this study.

## Abbreviations

The following abbreviations are used in this manuscript:

*L. japonica*	*Lonicera japonica* Thunb.
S1	The juvenile floral bud stage
S2	The three-green floral bud stage
S3	The two-white floral bud stage
S4	The complete white floral bud stage
S5	The silver flower stage
S6	The gold flower stage
S7	The withering flower stage
XP-CLR	Cross-population composite likelihood ratio
PCA	Principal component analysis
MeJA	Methyl jasmonate
LUC	Firefly luciferase
dCAPS	Derived cleaved amplified polymorphic sequence
MAS	Marker-assisted selection
