# Supplementary material for: Transcription Factor LjWRKY50 Affects Jasmonate-Regulated Floral Bud Duration in Lonicera japonica
Source: Plants (Basel). 2025 Jul 27;14(15):2328. doi: 10.3390/plants14152328 (PMC12349469; doi:10.3390/plants14152328)
Supplement: Supplementary file 1 [file plants-14-02328-s001.zip › Supplemental Figures.pdf]

Transcription Factor LjWRKY50 Affects Jasmonate-Regulated Floral Bud Duration in *Lonicera japonica*

Yanfei Li [1], Yutong Gan [1], Guihong Qi [1], Wenjie Xu [1], Tianyi Xin [1], Yuanhao Huang [1], Lianguo Fu [1], Lijun Hao [1], Qian Lou [1], Xiao Fu [2], Xiangyun Wei [2,3], Lijun Liu [3], Chengming Liu [3] and Jingyuan Song [1,*]

Supplemental Figure legend

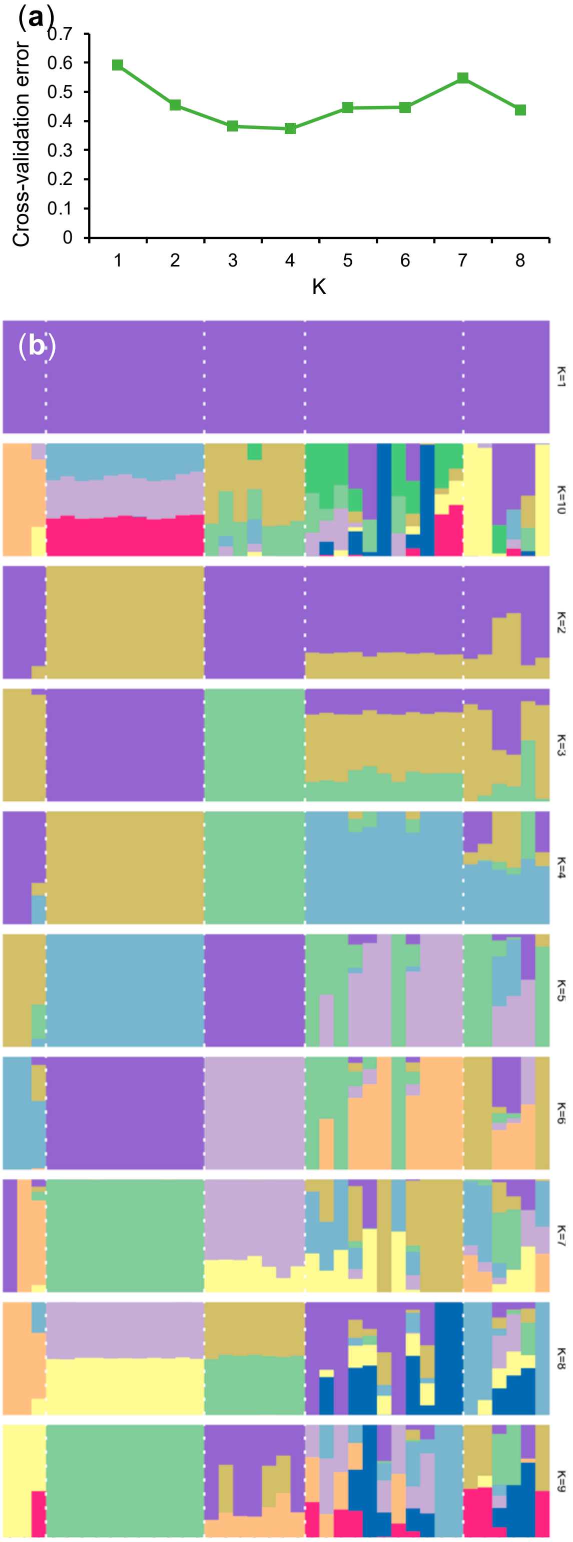

Figure S1 Population structure analysis of 38 Lonicera samples using ADMIXTURE software. (**a**) The cross-validation error from ADMIXTURE software; (**b**) Population

structure from clusters one to ten. Each color and bar represents an individual population and accession, respectively. The segments of different colors represent the proportions of ancestral populations. A dashed white line divides the accessions into five distinct clusters.

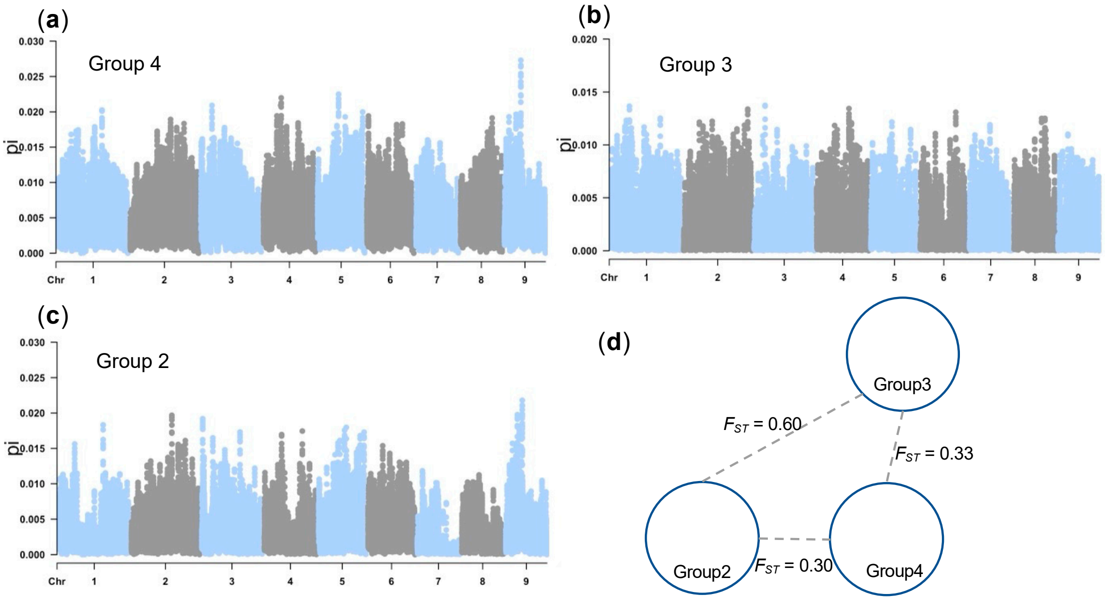

Figure S2 The nucleotide diversity (π) analyses using 35 resequenced *L. japonica* germplasms. (**a-c**) The Manhattan plots display the nucleotide diversity (π) of Group 4, Group 3, and Group 2 across the entire genome, employing a 100 kb window and a 10 kb step, respectively; (**d**) The average nucleotide diversity (π) within the group.

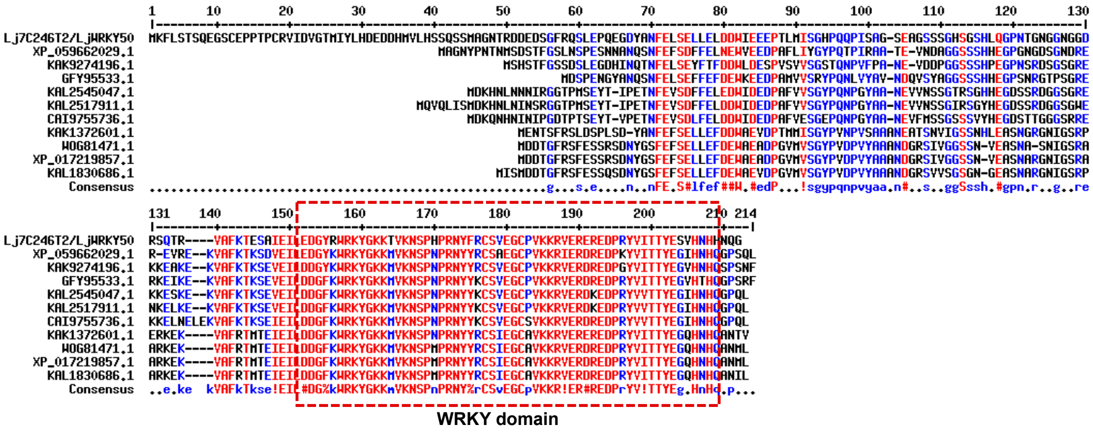

Figure S3 The alignment results of the putative protein sequence for WRKY50 in *L. japonica* and the other ten species.

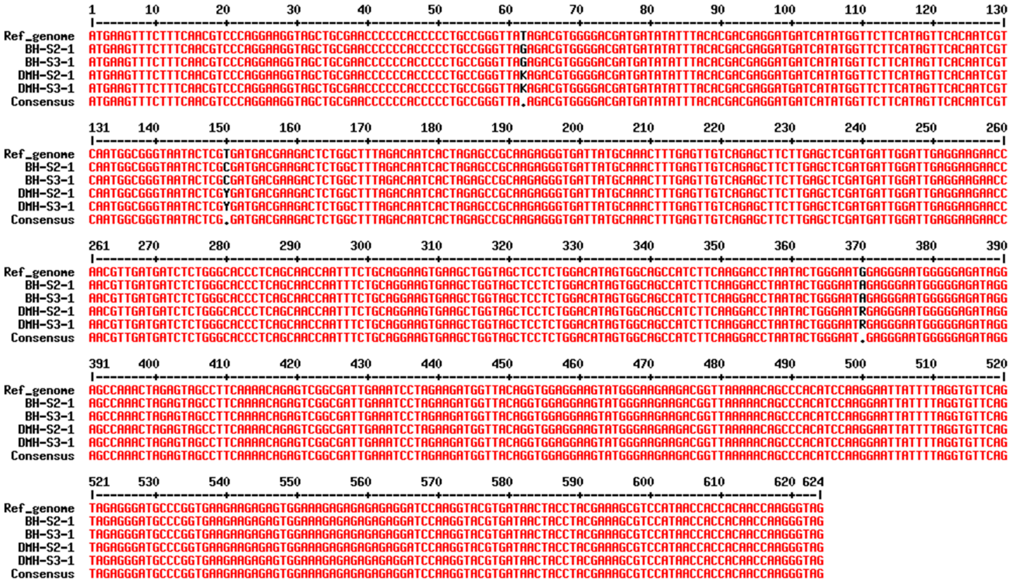

Figure S4 The alignment of CDS sequences for the LjWRKY50 gene, which was cloned from the long- and short-duration floral bud varieties BH and DMH, involved two biological replications.
